# Perspectives on medical 3D printing at the point-of-care from the new European 3D Printing Special Interest Group

**DOI:** 10.1186/s41205-022-00167-3

**Published:** 2023-05-05

**Authors:** Giovanni Biglino, Carina Hopfner, Joakim Lindhardt, Francesco Moscato, Josep Munuera, Gunpreet Oberoi, Alessandro Tel, Arnau Valls Esteve

**Affiliations:** 1 Bristol Medical School, University of Bristol, Bristol Royal Infirmary, Upper Maudlin Street, Bristol, BS2 8HW UK; 2 National Heart and Lung Institute, Imperial College London, London, UK; 3 Department of Pediatric Cardiology & Pediatric Intensive Care Medicine, LMU Klinikum, Munich, Germany; 4 3D Printing Center, Aarhus Universitetshospital, Aarhus, Denmark; 5 Center for Medical Physics and Biomedical Engineering, Medical University of Vienna, Vienna, Austria; 6 Ludwig Boltzmann Institute for Cardiovascular Research, Vienna, Austria; 7 Austrian Cluster for Tissue Regeneration, Vienna, Austria; 8 3D Unit (3D4H), Hospital Sant Joan de Déu, Universitat de Barcelona, Barcelona, Spain; 9 Department of Diagnostic Imaging, Hospital Sant Joan de Déu, Universitat de Barcelona, Barcelona, Spain; 10 Austrian Center for Medical Innovation and Technology in Vienna (ACMIT Gmbh), Wiener Neustadt, Austria; 11 University Hospital of Udine, Head & Neck and Neuroscience Department, Clinic of Maxillofacial Surgery, Udine, Italy; 12 Innovation in Health Technologies, Institut de Recerca Sant Joan de Déu, Esplugues de Llobregat, Spain; 13 Departament de Medicina i Recerca Translacional, Facultat de Medicina i Ciències de la Salut, Universitat de Barcelona, Barcelona, Spain

## Abstract

This editorial presents the vision for the newly formed (2022) European 3D Special Interest Group (EU3DSIG) in the landscape of medical 3D printing. There are four areas of work identified by the EU3DSIG in the current landscape, namely: 1) creating and fostering communication channels among researches, clinicians and industry, 2) generating awareness of hospitals point-of-care 3D technologies; 3) knowledge sharing and education; 4) regulation, registry and reimbursement models.

## Introduction

The European 3D Special Interest Group (EU3DSIG) was formed in June 2022 during the annual “3D Printing in Hospitals” meeting hosted in Leuven (Belgium) by Materialise (Leuven, Belgium) and met officially for the first time on December 2–3, 2022 at the M3d + it “Additive Manufacturing in Medicine” symposium hosted by the Medical University of Vienna and the Austrian Center for Medical Innovation and Technology in Vienna (Wiener Neustadt, Austria). In a programme stacked with presentations from experts in the field of medical 3D- and bioprinting addressing applications ranging across all areas of medicine (and veterinary) and covering both technical, translational and clinical aspects, the EU3DSIG presented in a dedicated session its mission and visions for advancing this field.

## The landscape

**Fig. 1:**
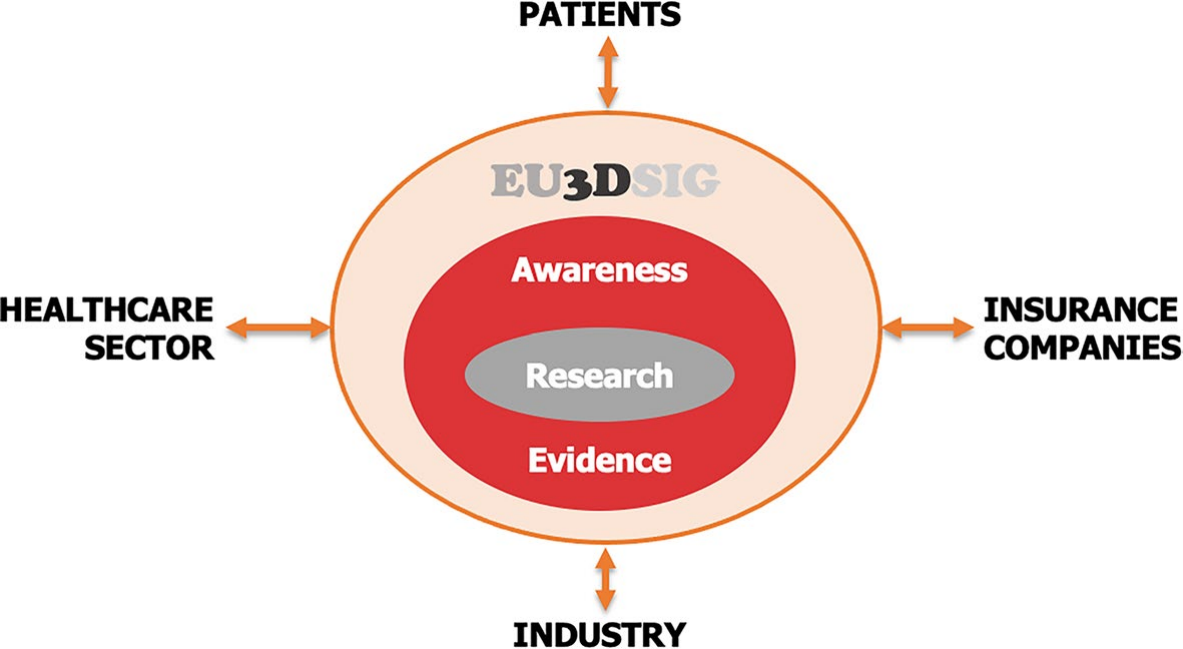
A vision for the European 3D Special Interest Group (EU3DSIG), with evidence and awareness rooted in research and aiming to foster a dialogue amongst relevant stakeholders.

Recognising the fast growth of the 3D Printing (3DP) at the point-of-care and the use of 3DP in hospitals all over the world, the mission of the EU3SIG is to promote excellence in healthcare outcomes, education, research and technological innovation using 3D technologies. The group aims to create a European community sharing knowledge and generating awareness with the clear focus on in-hospital 3D technologies, and to promote the use of 3D technologies at the point-of-care and thus advancing the quality of patient care via knowledge sharing, international projects and research. See Fig. 1. It is in the spirit of utmost collaboration with the existing important networks (chiefly the Radiological Society of North America, RSNA, 3D Printing Special Interest Group and the Society for Cardiovascular Magnetic Resonance, SCMR, 3D+ Advanced Visualization Special Interest Group) and industry that the EU3DSIG intends to contribute to the field.

The EU3DSIG shall address the current landscape characterized by an uncertain classification of 3D printed solutions as medical devices on the regulatory front, the lack of clarity about reimbursement in different European contexts, and the emergence of a recognised field of research requiring new skills and professional profiles. In this context, there are four areas of work that have been identified by this EU3DSIG:

1. Creating and fostering community: promoting ad-hoc communication channels among researches, clinicians and industry to foster an inclusive space where to share knowledge and informing collaborative frameworks in the medical 3DP ecosystem
2. Awareness of hospitals point-of-care 3D technologies: generating awareness around the impact of 3D technologies in medical applications through the participation in and organization of congresses, the active dissemination of related data through joint publications, building scientific evidence for point-of-care 3D technologies through multicentre studies including systematic reviews and metanalyses, and liaising with key stakeholders (scientific societies, hospitals, public engagement)
3. Knowledge sharing and education: structuring existing training and educational programs with focus on 3D technologies and resources across the European landscape and facilitating exchanges across 3D centres
4. Regulation, registry and reimbursement models: to take an evidence-based approach in coordinating initiatives around regulation and reimbursement, and informing future reimbursement models.

## Priorities

Beyond recognizing and supporting basic research advances in the field, such as 4D printing including the incorporation of mechanical meta-materials [1] or latest advances in 3D bioprinting (e.g. Freeform Reversible Embedding of Suspended Hydrogels or FRESH technology [2], the SIG will foster clinical, ideally multicenter, studies about applied research areas of 3D technologies in healthcare, including:

- Clinical uses of 3D-printed patient-specific models
- Device testing and research studies involving 3D-printed anatomical models (not necessarily patient-specific)
- The use of machine learning and artificial intelligence methodologies in the context of 3D applications
- Visualisation methodologies moving beyond the haptic features of 3DP (e.g. virtual reality, augmented reality, holography)
- 3D printing materials
- 3D printing of non-anatomical parts
- Optimization of imaging techniques involved in 3D technologies (from acquisition to segmentation)
- 3D bioprinting
- 3D printing of pharmaceuticals

On the medical educational front, whilst the consensus and current evidence suggest a valuable use of 3DP models across domains of knowledge retention and educational engagement, there is a recognised need for larger studies including longitudinal assessments to explore benefits over time when presenting students and trainees with 3D models [3, 4]. Similar observations apply to the use of models for facilitating communication with patients and improving patient experience, where current evidence is scarce and longitudinal data is entirely lacking [5, 6]. In general, a need for data-driven appropriateness has been discussed, including considerations of the technical needs for each clinical indication [7], and collaborative research efforts could help substantially in generating these data.

On the regulatory front, the European landscape for medical 3DP involves referring to the Medical Device Regulation (2017/745, the MDR) and the in vitro diagnostic medical regulation (2017/746, the IVDR), both applying directly in EU member states [8]. The MDR changes the definition of “custom-made” devices (i.e. not including mass-produced by means of industrial manufacturing processes in accordance with the written prescriptions of any authorised person) leaving a grey zone as to the interpretation of “mass-produced”, but also identifying another important domain in which a 3D printing lab could provide solutions manufactured on-site, within a hospital. Also, the UK’s Medicines and Healthcare products Regulatory Agency (MHRA) issued guidance on 3D printing medical devices particularly following the rise in 3D printing of personal protective equipment (PPE) during the Covid-19 pandemic, but also including custom-made medical devices (patient-specific) and distinguishing medical devices (regulated under the Medical Devices Directive/Regulations) and PPE (regulated under the PPE Regulation 2016/425). This guidance essentially indicates that the final determination is made by the manufacturer according to the device’s intended purpose, and that irrespectively all 3DP products should be CE-marked [9, 10].

In a rapidly evolving field, educational needs for professionals and opportunities for career development are also evolving, and whilst several courses for medical 3D printing are available offered by educational institutions but also industry leaders, the offering keeps changing. Whilst educational opportunities so far tended to be in the format of short courses (1–3 days), new models are emerging, e.g. a recent Medical 3D Printing Specialist certificate option for registered radiologic technologists interested in applying 3D printing technology (duration 2–4 semesters) or a hybrid postgraduate programme in image processing and 3D printing accredited by polytechnic and medical universities with ECTS credits, as a complementary acquisition of knowledge and skills to increase the level of expertise and technical-clinical specialization [11, 12], (https://www.clarksoncollege.edu/radiography-medical-imaging/degree-options/medical-3d-printing-specialist-certificate/index, http://www.interacsalut.cat/index.php?page=gw&gw_idp=61&lang=en/2020-2021). This leads to considering education standards in the near future, potentially, or requirements for newly emerging professional roles, as well as questions about potential future inclusions within the medical curriculum in different forms.

## Beware of technological hypes

Coordination across centres, ensuring constructive communication both with industrial players in this field but also with patients who may benefit from all ranges of 3DP devices, can result in expanding the current evidence-base with large multicentric studies and provide clarify around regulations and models for reimbursement. This is where the EU3DSIG intends to position itself. One interesting point emerged during the M3d + it meeting in Vienna concerning the technological hype. Whilst recognising that medical applications of 3D technologies now date over 30 years when thinking of early pioneering work in the late 1980s/early 1990s, the field has hugely expanded particularly over the past decade, but in our technology-driven (arguably technology-obsessed) society the novelty factor expires quickly, sometimes resulting in efforts (be it research or social engagement or cross-sector partnerships) in all good faith focused on the next novel technological gadget. Whilst advances in the context of all 3D+ technologies are incredibly exciting (first and foremost all visualisation opportunities offered by virtual, augmented and mixed reality), it should be recognised that more applied and translational research is still needed in medical 3DP. This includes also systematic evaluation of cost-benefit analyses in different settings and impact evaluation clinical trials.

*Note*: if you would like to find out more about the EU3DSIG and future events organised by the group, or to get in touch, please visit: https://www.linkedin.com/company/eu3dsig/

## Data Availability

Not applicable.
